# Society Meetings

**Published:** 1906-07

**Authors:** 


### SOCIETY MEETINGS.

The Central Medical Association of Lancaster and vicinity at its regular meeting held Tuesday evening, May 22, 1906, took up the subject of contract work by physicians and thoroughly discussed it. It was agreed that no physician in Lancaster or Depew should act as contract physician for any fraternal organisation, society or corporation for any specified sum or per capita fee. This is intended to apply to contracts for medical treatment or surgical operations.

The Buffalo Academy of Medicine held its annual meeting June 12, 1906, at which the following named officers were elected for the ensuing year: president, Charles Sumner Jewett; treasurer, William Irving Thornton; trustee, Grover W. Wende. Dr. Herbert U. Williams, the retiring president, delivered his address, after which an informal supper was served at the Hotel Lafayette.

The American Laryngological Association held its twenty-eighth annual meeting at Niagara Falls, May 31 - June 2, 1906. Dr. W. Scott Renner, of Buffalo, entertained the members and guests at luncheon on the first day of the meeting.

The Medical Societies of the Counties of Alleghany and Cattaraugus, N. Y., and of McKean, Pa., will hold a joint meeting at Rock City, July 17 and 18, 1906, at which Dr. Roswell Park of Buffalo will deliver an address.

The Lake Keuka Medical and Surgical Association will hold its seventh annual meeting at Grove Springs, Lake Keuka, July 5, 6, and 7, 1906, under the presidency of Dr. W. B. Jones, of Rochester. An elaborate program has been prepared which contains the names of Drs. J. F. W. Whitbeck, of Rochester, X. O. Werder, of Pittsburg, and George W. Crile, of Cleveland, who will present papers. Dr. H. B. Nichols, of Pulteney, is the secretary.

